# Curcumin Opposes Perfluorodecanoic Acid–Induced Adipogenesis Through the PPARγ/C/EBPα Axis in 3T3-L1 Cells

**DOI:** 10.3390/toxics14090741

**Published:** 2026-08-22

**Authors:** Ayten Darcan, Ezgi Nur Çil, Yasemin Soysal

**Affiliations:** 1 Institute of Health Sciences, Department of Molecular Medicine, Dokuz Eylül University, 35340 İzmir, Türkiye; aytendrcn@gmail.com (A.D.); ezgi.cil@adu.edu.tr (E.N.Ç.); 2 Faculty of Health Sciences, Department of Nutrition and Dietetics, Aydın Adnan Menderes University, 09010 Aydın, Türkiye

**Keywords:** adipogenesis, curcumin, obesogen, perfluorodecanoic acid (PFDA), perfluoroalkyl substance, PPARγ, 3T3-L1, gene expression

## Abstract

(1) **Background**: Perfluorodecanoic acid (PFDA), a persistent perfluoroalkyl obesogen, promotes adipogenesis, yet whether its effects can be pharmacologically opposed remains unknown. (2) **Methods**: We examined the interaction between PFDA (50, 75 μM) and the dietary polyphenol curcumin (20, 30 μM) throughout the 8-day 3T3-L1 preadipocyte differentiation protocol, using cell-viability (WST-1) assays with a two-factor interaction analysis, Oil Red O lipid quantification and mRNA profiling of *Pparg*, *Cebpa*, *Srebf1*, and *Fasn*. (3) **Results**: PFDA alone increased lipid accumulation and upregulated *Pparg*, *Cebpa*, and *Fasn*, whereas *Srebf1* was induced only weakly and only at the higher dose, a pattern compatible with preferential engagement of the PPARγ/C/EBPα program over the SREBP-1c lipogenic pathway at the mRNA level, although the transcripts were not formally compared and no protein-level data were obtained. At two sub-cytotoxic concentrations, curcumin preserved potent antiadipogenic activity, reducing lipid accumulation by up to 67.3% and reversing PFDA-induced gene upregulation. At the viability endpoint the two compounds acted independently: curcumin significantly reduced viability and PFDA significantly increased it, with no statistically detectable interaction between them (two-way ANOVA, interaction *p* = 0.85). (4) **Conclusions**: This dissociation shows that a chemical interaction defined at one biological endpoint does not predict its outcome at another, a principle with broad implications for mixture toxicology—and it establishes curcumin as a candidate dietary intervention that intercepts an obesogen’s transcriptional program against environmentally driven metabolic disruption.

## 1. Introduction

The obesity epidemic constitutes an escalating global health burden [1]. By 2035, projections indicate that approximately 4 billion people will be overweight or obese, a condition expected to reduce potential global economic income by over USD4 trillion [2]. Epidemiological and experimental studies show that environmental chemicals and food chemical contaminants affect metabolic homeostasis alongside genetic and lifestyle factors, increasing susceptibility to obesity and associated metabolic disorders [3]. Obesity arises centrally through adipogenesis, the process by which preadipocytes differentiate into mature adipocytes. Adipocyte differentiation depends mainly on two transcription factors: peroxisome proliferator-activated receptor γ (PPARγ) and CCAAT/enhancer-binding protein α (C/EBPα). These proteins activate adipocyte-specific genes that initiate and sustain terminal differentiation [4]. In addition to these master regulators, sterol-sensitive factors further modulate the lipogenic pathway. Sterol regulatory element-binding protein 1 (SREBP-1) is a positive regulator of adipogenesis that transcriptionally regulates fatty acid synthase (FASN), the primary enzyme for fatty acid biosynthesis and triglyceride accumulation. Its relationship with PPARγ is reciprocal rather than unidirectional: SREBP-1 enhances PPARγ activity both by inducing its expression and by generating endogenous PPARγ ligands, while PPARγ activation in turn upregulates SREBP-1 expression in differentiating adipocytes. This bidirectional coupling means that a change in either factor can propagate to the other, a consideration that bears directly on the interpretation of transcript-level data in the present study [5]. Inhibition of this differentiation process represents a critical therapeutic strategy against obesity-related metabolic dysfunction, and the 3T3-L1 preadipocyte cell line has long been established as an in vitro model for evaluating adipogenesis and, more recently, has been proposed as an alternative screening model for environmental obesogens [6].

Perfluoroalkyl and polyfluoroalkyl substances (PFASs) are widely used across industrial sectors as surface-treating agents for commodities such as paperboard packaging, carpets, leather goods, and textiles. Among PFAS compounds, perfluorodecanoic acid (PFDA) is among the least documented, and further investigation into its specific industrial roles and biological impacts is warranted [7]. Exposure to environmental chemicals and food contaminants, including synthetic perfluorinated compounds (PFCs), disrupts steroidogenesis and alters human metabolism, thereby predisposing individuals to weight gain and contributing to the development of obesity and metabolic disorders [8]. Perfluorodecanoic acid (PFDA, C_10_HF_19_O_2_, CAS No: 335-76-2), a toxic and persistent environmental pollutant, is widely used in manufacturing chemical coatings for food packaging and cookware. Perfluorinated compounds (PFCs), such as PFDA, are classified as endocrine disruptors and are positively associated with human obesity [9]. PFDA promotes adipogenesis in 3T3-L1 in a manner linked to activation of the PPARγ nuclear receptor, which cooperates with C/EBPα to drive adipocyte maturation [10]. Upregulation of lipogenic genes such as *Fasn* and *Srebf1* stimulates lipid metabolism and triglyceride accumulation in 3T3-L1 adipocytes [9]. Curcumin, a biologically active polyphenolic compound isolated from the rhizomes of *Curcuma longa*, exhibits antioxidant, anti-inflammatory, and antiadipogenic properties [11]. Research shows that curcumin suppresses adipogenesis in cell culture and animal models by downregulating PPARγ and C/EBPα pathways, thereby inhibiting the differentiation of 3T3-L1 preadipocytes into adipocytes [11]. In a closely related precedent, curcumin supplementation reduced *Pparg* and *Cebpa* mRNA levels and attenuated triglyceride accumulation in 3T3-L1 cells exposed to benzyl butyl phthalate, another food-contact endocrine disruptor [3].

Human exposure to PFDA occurs mainly via the gastrointestinal tract, from both direct dietary intake and in vivo biotransformation of precursor compounds, with diet and drinking-water quality being the primary factors influencing internal PFAS burden [12,13,14]. Recent NHANES biomonitoring data cohorts report median serum PFDA concentrations of approximately 0.2 ng/mL (~0.39 nM), and longitudinal datasets indicate a gradual decline in systemic burdens over the past two decades [15,16]. PFDA distributes into protein-rich tissues such as the liver, kidney and lung rather than partitioning into neutral lipid, owing to its long half-life and high affinity for serum albumin and fatty-acid-binding proteins. Reported human tissue-to-serum ratios are nevertheless modest, on the order of 1.3:1 for liver, and adipose tissue does not represent a preferential accumulation depot [17,18]. Its continued detection in cord blood and breast milk further underscores its persistence and potential for maternal-fetal transfer [19]. Adipose tissue is therefore relevant to PFDA toxicology as the site at which adipogenesis occurs rather than as a high-accumulation reservoir, establishing the 3T3-L1 adipocyte as a mechanistically appropriate model for interrogating its adipogenic activity.

Although PFDA is increasingly recognized as a potential metabolic disruptor, no study has examined whether pharmacological intervention can oppose its pro-adipogenic activity or identified the transcriptional nodes through which such opposition might occur. This study was designed to address this gap. Having first characterized the effects of PFDA exposure on adipogenesis and lipogenesis in 3T3-L1 cells, we subsequently evaluated whether curcumin co-treatment counteracts the associated adipogenic and lipogenic activity. We examined two functionally distinct arms of the adipogenic program to resolve the mechanistic basis of this interaction: the master transcriptional axis governed by *Pparg* and *Cebpa*, and the lipogenic axis represented by *Srebf1* and its downstream effector *Fasn*. By mapping how these four genes respond to single-agent and combination exposures, we addressed two questions: whether curcumin attenuates PFDA-driven adipogenesis and where in the transcriptional hierarchy this opposition occurs. This approach separates targets that PFDA engages directly from those that curcumin modulates on its own.

## 2. Materials and Methods

### 2.1. Materials

Curcumin (Santa Cruz Biotechnology, Dallas, TX, USA), PFDA (Thermo Fisher Scientific, Waltham, MA, USA, CAS: 335-76-2, 97%, Catalog number: B22292.06), dimethyl sulfoxide (DMSO) (Santa Cruz Biotechnology, Dallas, TX, USA), 3-isobutyl-1-methylxanthine (IBMX) (I5879, Sigma-Aldrich, St. Louis, MO, USA), insulin (INS) (I0516, Sigma-Aldrich, St. Louis, MO, USA), dexamethasone (DEX) (D4902, Sigma-Aldrich, St. Louis, MO, USA), DMEM (Euroclone S.p.A., Pero, Milan, Italy, ECM0103L), fetal bovine serum (FBS) (Capricorn Scientific GmbH, Ebsdorfergrund, Germany, FBS-HI-11A) and penicillin/streptomycin (Gibco/Thermo Fisher Scientific, Waltham, MA, USA, 15140122) were all of analytical grade.

### 2.2. 3T3-L1 Cell Culture and Maintenance

3T3-L1 mouse preadipocytes provided by the American Type Culture Collection (ATCC, CL-173, Manassas, VA, USA) were maintained in high-glucose DMEM supplemented with 10% FBS and 100 U/mL penicillin/100 μg/mL streptomycin at 37 °C under a 5% CO_2_ atmosphere. Cells were passaged at 70–80% confluency to avoid contact inhibition and maintain differentiation potential. For cryopreservation, cells were suspended at a density of 1 × 10^6^ cells/mL in DMEM containing 10% DMSO and stored at −80 °C first and then transferred to liquid nitrogen for long-term storage. All experiments were conducted with cells at a similar low passage (≤8th passage) [6,20].

### 2.3. Differentiation Protocol

For differentiation, cells were seeded at approximately 10–15 × 10^4^ cells per well in 6-well culture plates. Differentiation commenced on Day 0, two days after the cells achieved 100% confluency, by switching to differentiation medium 1 (DF1). This medium consisted of DMEM supplemented with 10% FBS, 2 mM L-glutamine (i.e., 1% of a 200 mM stock) (Gibco), 100 U/mL penicillin/100 μg/mL streptomycin, 0.5 mM IBMX, 1 μM dexamethasone (DEX), and 1 μg/mL insulin. The medium was replaced on Day 2 with differentiation medium 2 (DF2), consisting of DMEM supplemented with 10% FBS, 1 μg/mL insulin, 2 mM L-glutamine, 100 U/mL penicillin/100 μg/mL streptomycin, and the cells were maintained in this environment for 48 h. From Day 4 onward, cells were maintained in 10% FBS/DMEM supplemented with 2 mM L-glutamine and 100 U/mL penicillin/100 μg/mL streptomycin, with media replaced every 48 h until full adipocyte maturation was achieved by Day 8. All experiments included a DMSO-only vehicle control consisting of 0.1% DMSO in DF1 [6,20]. Consistent with the report by Zhao et al. [21], we observed a decline in differentiation efficiency following repeated freeze–thaw cycles, and found that subconfluent passaging was critical for preserving adipogenic capacity. These cell line-specific characteristics were carefully managed throughout the study.

### 2.4. Cell Viability Assay

Cell viability was evaluated using the WST-1 assay (Cayman Chemical, Ann Arbor, MI, USA, 10008883) according to the manufacturer’s protocol. Cells were seeded at approximately 6 × 10^3^ cells per well in 96-well plates and grown to 100% confluency and differentiated by adipocyte differentiation medium. At the same time curcumin and PFDA treatments were initiated on Day 0, coinciding with the onset of differentiation, and viability was assessed after a 48 h incubation as a dose-finding readout. Cells were then treated with WST-1 reagent (10 μL/well) after exposure to a differentiation medium containing curcumin (25–200 μM), PFDA (50–500 μM), or their combinations. Plates were incubated at 37 °C and 5% CO_2_ for 2 h, after which absorbance at 450 nm was measured using an ELISA plate reader (Synergy HT, BioTek Instruments, Winooski, VT, USA). Experiments were performed with five technical replicates per biological replicate (*n* = 3). Cell viability was expressed relative to the DMSO vehicle control (0.1% DMSO), which was defined as 100% viability, and calculated using the formula: % Viability = (Mean Sample Absorbance/Mean Control Absorbance) × 100. The IC_50_ was calculated by nonlinear regression, and doses below this threshold were selected for subsequent experiments. Data processing and graph creation were performed using GraphPad Prism 10 (GraphPad Software, Inc., La Jolla, CA, USA). The combination of curcumin and PFDA was initially considered for analysis by the Chou–Talalay median-effect method [22], but this approach proved inapplicable to the present compound pair. The method requires each single agent to display a monotonic dose–effect relationship and to produce a measurable effect at the concentrations combined, so that an equi-effective single-agent dose can be determined. Neither condition was met by PFDA, which produced a hormetic, non-monotonic response with viability exceeding the vehicle control between 75 and 300 μM and cytotoxicity confined to 400–500 μM, and which was not cytotoxic at either concentration used in combination The interaction between the two compounds was therefore evaluated on a two-factor basis, which the design supports because the complete curcumin (0, 20, 30 μM) by PFDA (0, 50, 75 μM) matrix, including the vehicle control and both single agents, was assayed on a single plate. For each combination the observed viability was compared with the viability expected in the absence of interaction, and the difference between the two was taken as the interaction term, a positive value indicating less-than-additive and a negative value greater-than-additive cytotoxicity. Two independent reference models were applied so that the conclusion would not rest on a single assumption about how the two agents combine. Under the Bliss independence model, which assumes that the compounds act through independent mechanisms, the expected viability was calculated as V(expected) = (V(A) × V(B))/100, where V(A) and V(B) are the viabilities of each agent alone expressed as a percentage of the vehicle control [23]. Under a response-additivity model, the expected viability was calculated as V(expected) = V(A) + V(B) − 100, that is, the sum of the individual changes from control. The two models make different assumptions and were therefore used in parallel as a consistency check. This analysis was applied to the cell-viability endpoint only, on which both compounds act in the same direction and can be expressed on a common scale; it was not applied to adipogenesis, on which the two agents act in opposition and for which the median-effect formalism is in any case inapplicable. Interaction terms are reported as quantitative estimates of synergy or antagonism. As a model-free alternative, the interaction was additionally evaluated on a two-factor basis using the complete curcumin (0, 20, 30 μM) by PFDA (0, 50, 75 μM) design assayed on a single plate, comparing observed combination viability with the additive expectation derived from the corresponding single-agent effects.

### 2.5. PFDA and Curcumin Exposure to Cells

PFDA and curcumin stock solutions were prepared at 250 mM in DMSO and subsequently diluted into growth/differentiation medium such that the final DMSO concentration did not exceed 0.2% (*v*/*v*), Because solvent load scales directly with final compound concentration, DMSO was 0.008% and 0.012% (*v*/*v*) for curcumin at 20 and 30 μM, 0.020% and 0.030% for PFDA at 50 and 75 μM, and, in the combination groups, 0.028% (20 μM curcumin + 50 μM PFDA), 0.038% (20 μM + 75 μM), 0.032% (30 μM + 50 μM) and 0.042% (30 μM + 75 μM). The DMSO vehicle control contained 0.1% (*v*/*v*) and therefore carried approximately 2.4-fold more solvent than any treatment group in the differentiation and combination experiments, representing a conservative worst-case solvent exposure. DMSO rose to 0.12–0.20% (*v*/*v*) only at 300–500 μM PFDA in the initial cytotoxicity screen; these concentrations were used for range-finding only and in no differentiation, lipid-accumulation or gene-expression experiment. The solutions were sterilized by filtration through a 0.22 μm filter (Biosharp Life Sciences, Hefei, Anhui, China) and subsequently stored at −20 °C. Cell viability was assessed after 48 h of treatment with the specified concentrations of PFDA and curcumin, both initiated on Day 0 in accordance with the cell differentiation protocol described above [3,9,11]. Dose-trial experiments on cell viability and lipid accumulation yielded selections of 50 μM and 75 μM PFDA, alongside 20 μM and 30 μM curcumin (cu.), for incorporation into the differentiation medium. Based on the IC_50_ values and Oil Red O staining results, the following combinations were selected as experimental doses: 20 μM curcumin + 50 μM PFDA, 20 μM curcumin + 75 μM PFDA, 30 μM curcumin + 50 μM PFDA and 30 μM curcumin + 75 μM PFDA.

### 2.6. Oil Red O Staining for Lipid Accumulation

Oil Red O (ORO) staining was used to measure intracellular lipid accumulation on Day 8. A 0.5% stock solution of Oil Red O (O0625, Sigma-Aldrich, St. Louis, MO, USA) was prepared by dissolving 0.2 g of powder in 40 mL of isopropanol. The working solution was prepared fresh by diluting the stock 3:2 with distilled water, then filtering through a 0.22 μm membrane. Cells were fixed in 4% paraformaldehyde (pH 7.4) for either 45 or 60 min, rinsed with 60% isopropanol, stained with Oil Red O working solution for 15 min, and then washed with distilled water. For quantitative measurement, the stain was eluted with 100% isopropanol for 5 min. Absorbance at 510 nm was then measured with a microplate reader (Synergy HT, BioTek Instruments, Winooski, VT, USA) [24,25].

### 2.7. RNA Isolation

Total RNA was isolated on Day 8 of differentiation, when intracellular lipid content was maximal. Culture medium was removed, cells were washed twice with ice-cold 1× PBS, and 500 μL NucleoZOL reagent (Macherey-Nagel GmbH & Co. KG, Düren, Germany; Catalog number: 740404.200) was added per well. Following the addition of 200 μL nuclease-free water and vortexing, samples were centrifuged at 12,000× *g* for 15 min at 4 °C. The aqueous supernatant was transferred, mixed with an equal volume of 100% isopropanol, incubated at room temperature for 10 min, and centrifuged again at 12,000× *g* for 10 min at 4 °C. The RNA pellet was washed twice with 75% ethanol, air-dried, and resuspended in 20 μL nuclease-free water. RNA quantity and purity were assessed using a NanoPhotometer^®^ NP80 (Implen GmbH, Munich, Germany), and samples were stored at −80 °C.

### 2.8. cDNA Synthesis

Complementary DNA (cDNA) was synthesized from isolated RNA using a commercial reverse transcription kit (OneScript Plus cDNA Synthesis Kit, Applied Biological Materials Inc. (abm), Richmond, BC, Canada; Cat. No. G236) according to the manufacturer’s protocol. Each reaction (20 μL total volume) contained 1000 ng total RNA, 4 μL 5× RT Buffer, 1 μL dNTPs, 1 μL random primers, 1 μL reverse transcriptase, and nuclease-free water. Thermal cycling was performed on a T100 thermal cycler (Bio-Rad Laboratories Hercules, CA, USA) with the following conditions 50–55 °C for 15 min, 85 °C for 5 min, then held at 4 °C and cDNA was stored at −20 °C.

### 2.9. Measurement of the mRNA Expression Levels of Gapdh, Pparg, Cebpa, Srebf1, and Fasn

Expression levels of *Gapdh*, *Pparg*, *Cebpa*, *Srebf1*, and *Fasn* were measured by RT-qPCR using a commercial master mix (Applied Biological Materials Inc., (abm), Richmond, BC, Canada; Cat. No. G891). Quantitative PCR was performed on a Roche LightCycler 480 (Roche Diagnostics, Rotkreuz, Switzerland) qPCR machine with Software (v.1.5; Roche) on day 8 of adipocyte differentiation. Gene-specific primers were designed using NCBI BLAST+2.15.0. Each 20 μL reaction contained 100 ng cDNA, 0.5 μL of forward primer and 0.5 μL of reverse (both 10 μM), 10 μL BlasTaq 2 × qPCR MasterMix (G891, Applied Biological Materials Inc.) with nuclease-free water. The annealing temperature was 60 °C for *Pparg* and *Cebpa*, and 63 °C for *Srebf1* and *Fasn*. Relative gene expression was calculated using the 2^−ΔΔCt^ method. All data derive from three independent biological replicates (*n* = 3), each measured in technical triplicate. The primer sequences for *Gapdh*, *Pparg*, *Cebpa*, *Srebf1*, and *Fasn* were as follows: *Gapdh* forward 5′-TCCCACTCTTCCACCTTC-3′, reverse 5′-CTGTAGCCGTATTCATTGTC-3′; *Pparg* forward 5′-TCAGCTCTGTGGACCTCTCC-3′, reverse 5′-ACCCTTGCATCCTTCACAAG-3′; *Cebpa* forward 5′-CAAGAACAGCAACGAGTACCG-3′, reverse 5′-GTCACTGGTCAACTCCAGCAC-3′; *Srebf1* forward 5′-CGACTACATCCGCTTCTTGCAG3′, reverse 5′-CCTCCATAGACACATCTGTGCC-3′; and *Fasn* forward 5′-CACAGTGCTCAAAGGACATGCC-3′, reverse 5′-CACCAGGTGTAGTGCCTTCCTC-3′. *Gapdh* served as the reference gene because prior 3T3-L1 adipogenesis studies using identical differentiation and treatment conditions had already employed it to normalize expression of the same adipogenic targets examined in this work [3,11]. The coefficient of variation of raw *Gapdh* Ct values remained consistently low across all qPCR plates despite the use of a single reference gene (mean CV = 2.6%, range 1.6–3.9%), reflecting minimal technical variability in reference-gene expression. To further minimize differentiation-associated variation, all treatment groups, including the DMSO vehicle control, were harvested at a single fixed differentiation endpoint (Day 8) and processed in parallel under identical conditions within each run.

### 2.10. Statistical Analysis

All data are reported as mean ± standard deviation and are based on three independent biological replicates (*n* = 3). Within each biological replicate, technical wells were averaged before analysis, so that all inference is performed at the level of biological replication. For cell-viability (WST-1) and lipid-accumulation (Oil Red O) data, each biological replicate was normalized to its own DMSO vehicle control before analysis in order to remove between-run differences in baseline signal. Differences from the vehicle control were evaluated by one-way ANOVA with Dunnett’s post hoc test. Planned comparisons between each PFDA-alone group and its corresponding combination group, and between each curcumin monotherapy and its corresponding combination, were made using paired *t*-tests. The interaction between curcumin and PFDA at the viability endpoint was assessed in two ways: by two-way ANOVA on the complete curcumin (0, 20, 30 μM) × PFDA (0, 50, 75 μM) design, with biological replicate included as a blocking factor; and by comparing the observed combination viability with the expectation from two reference models, Bliss independence and response additivity, as described in Section 2.4.

Because 2^−ΔΔCt^ fold-change values are log-normally distributed, relative gene-expression data were analysed on the log2 scale: one-sample *t*-tests against a null value of 0 (i.e., a fold change of 1) for comparisons with the DMSO control, and two-sample *t*-tests for comparisons between PFDA-alone and combination groups.

Statistical significance was defined as * *p* < 0.05, ** *p* < 0.01, *** *p* < 0.001, and **** *p* < 0.0001. Analyses were performed using GraphPad Prism 10 (GraphPad Software Inc., La Jolla, CA, USA) and Python 3.12 (Python Software Foundation, Wilmington, DE, USA) with the SciPy (version 1.14) and statsmodels (version 0.14) packages.

## 3. Results

### 3.1. Cell Viability and Dose Selection

#### 3.1.1. Effect of Curcumin and PFDA on Cell Viability

**Figure 1:**
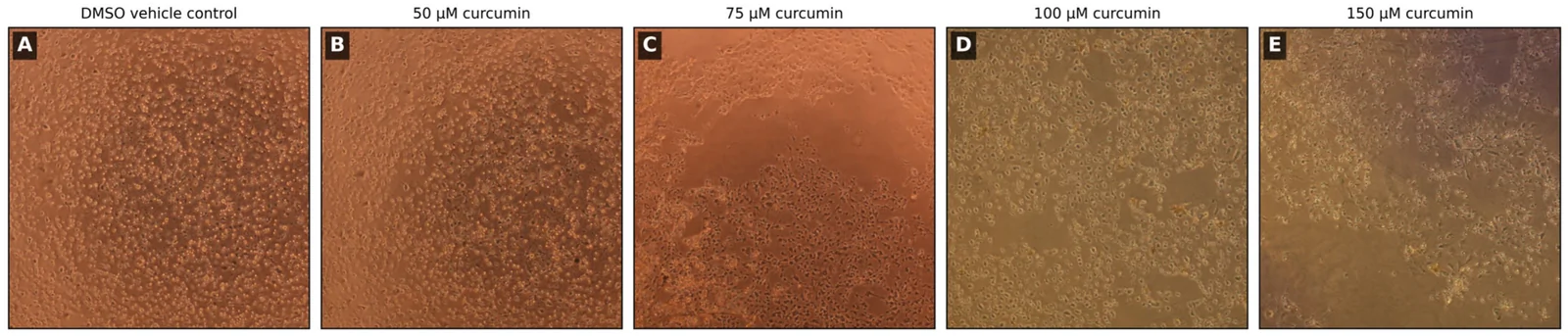
Phase-contrast micrographs of 3T3-L1 cells cultured in 6-well plates, captured 48 h after treatment with increasing concentrations of curcumin at 4× magnification. (**A**) DMSO vehicle control; (**B**–**E**) curcumin at 50, 75, 100, and 150 μM. Cell density and monolayer integrity are preserved at 50 μM (**A**,**B**). At 75 μM (**C**) the monolayer becomes discontinuous, with cell-free areas appearing, and at 100 and 150 μM (**D**,**E**) cell density is further reduced. Together with the concentration-dependent decline in WST-1 viability shown in Figure 1A, these observations support the selection of 20 μM and 30 μM as sub-cytotoxic working concentrations, which lie below the lowest concentration at which morphological changes were detected.

**Figure 2:**
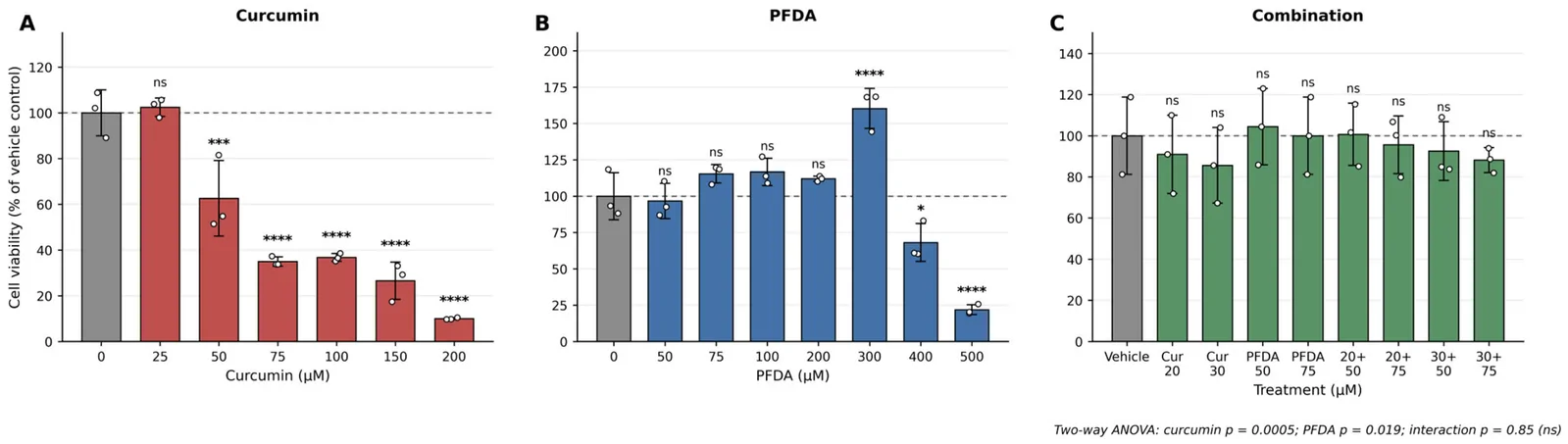
Effect of curcumin, PFDA and their combination on 3T3-L1 cell viability. Viability was assessed by WST-1 assay and is expressed as a percentage of the DMSO vehicle control (dashed line). (**A**) Curcumin alone, 25–200 μM. (**B**) PFDA alone, 50–500 μM. (**C**) Single agents and their combinations. Bars represent mean ± SD of three independent biological replicates (*n* = 3); open circles show the individual biological replicate values, each the mean of five technical wells. Statistical comparisons against the vehicle control were made by one-way ANOVA with Dunnett’s post hoc test: ns, not significant; * *p* < 0.05; *** *p* < 0.001; **** *p* < 0.0001. In (**C**), the curcumin × PFDA interaction was additionally assessed by two-way ANOVA.

To determine safe working concentrations, 3T3-L1 cells were exposed to increasing curcumin concentrations for 48 h and viability was assessed by WST-1 assay. Curcumin exhibited no cytotoxicity at 25 μM relative to the DMSO vehicle control. At 50 µM, viability decreased significantly to 62.8% (*p* < 0.0001), and declined further at higher concentrations, reaching 34.9% at 75 μM, 36.5% at 100 μM, 26.4% at 150 μM and 10.0% at 200 μM. Phase-contrast examination of parallel cultures was consistent with these measurements, showing altered cell morphology and reduced adherence at concentrations of 50 μM and above (Figure 1). The IC_50_ value was 56.15 μM. Both 20 μM and 30 μM were selected as working concentrations for subsequent experiments, as each remained below the cytotoxic threshold while retaining inhibitory activity relevant to adipogenic endpoints (Figure 2).

**Figure 3:**
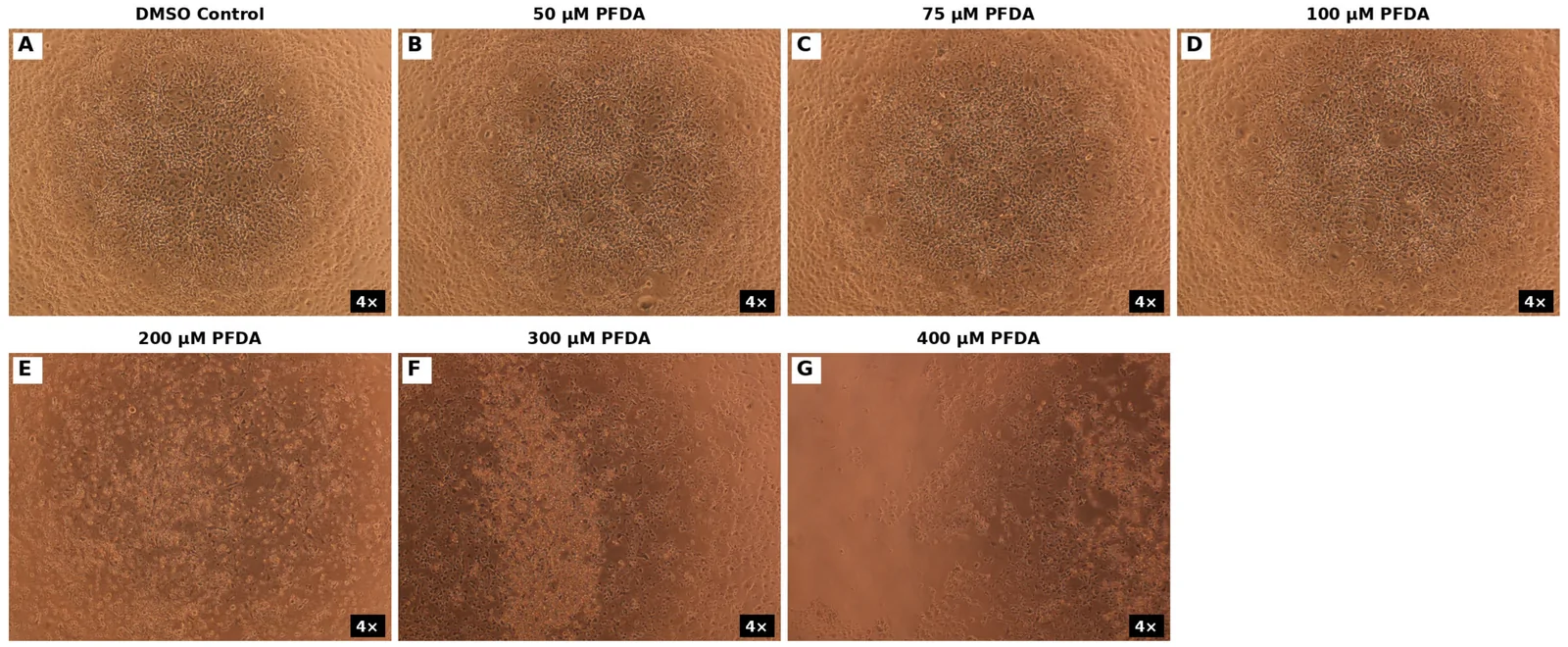
Phase-contrast micrographs of 3T3-L1 cells cultured in 6-well plates, captured 48 h after treatment with increasing concentrations of PFDA at 4× magnification. (**A**) DMSO vehicle control; (**B**–**G**) PFDA at 50, 75, 100, 200, 300, and 400 μM. Cell morphology and confluency remain intact at concentrations up to 100 μM (**A**–**D**). Concentrations of 200 μM or higher induce progressive cell rounding, detachment, and a loss of confluency (**E**–**G**). At concentrations ≥200 μM in the 6-well format, PFDA exhibited morphological cytotoxicity below the ~418 μM nominal cytotoxicity threshold estimated from the 96-well WST-1 assay (a value that, given the non-monotonic profile, is not a conventional IC_50_); this justified the conservative selection of 50 μM and 75 μM working concentrations to preserve morphological integrity.

**Figure 4:**
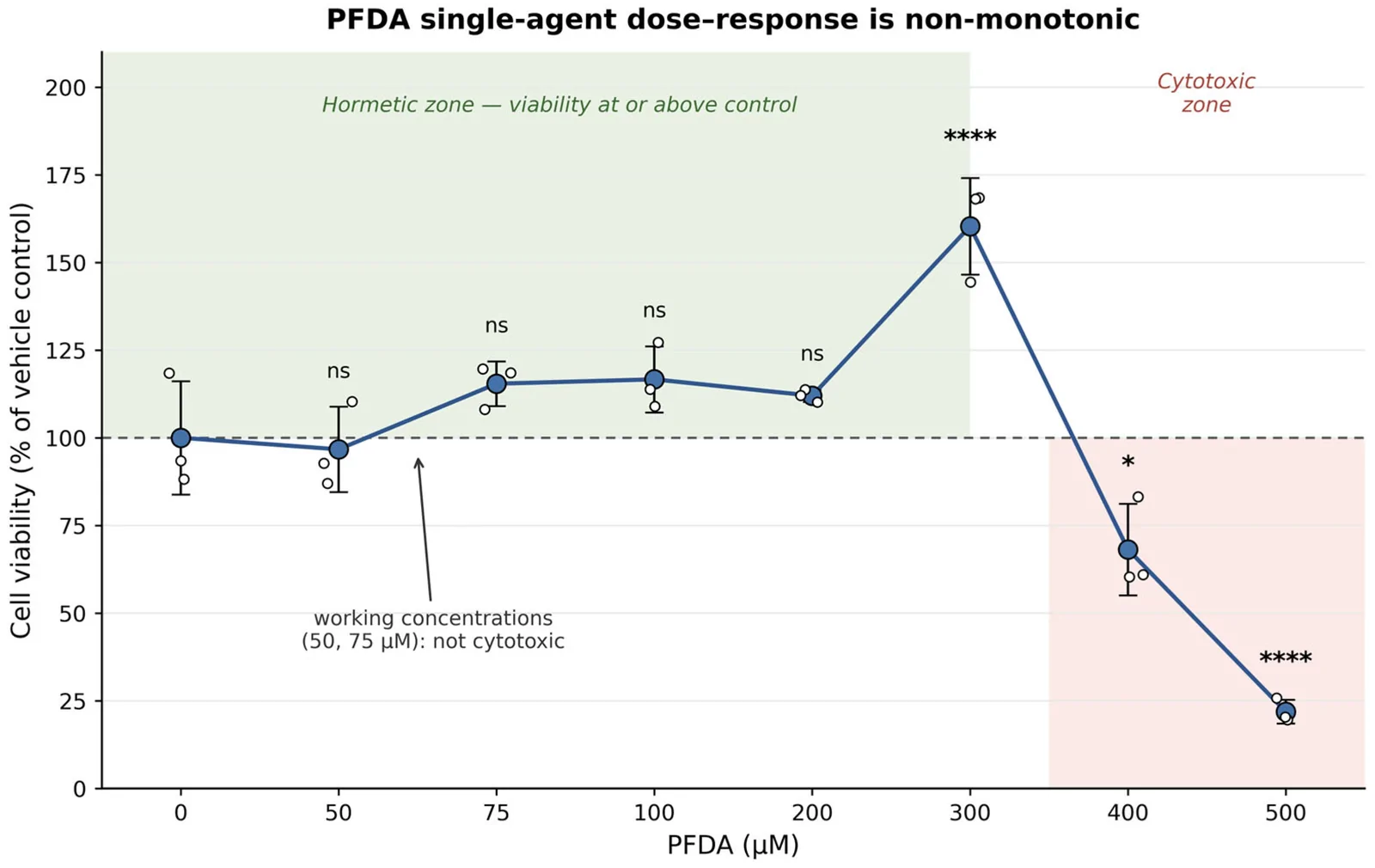
Non-monotonic single-agent dose–response of PFDA in 3T3-L1 cells (WST-1 assay), presented in expanded form to support the methodological argument in Section 2.4. Viability is expressed as a percentage of the DMSO vehicle control (dashed line); points show mean ± SD of three independent biological replicates (*n* = 3), with open circles indicating individual biological replicate values, each the mean of five technical wells. Shaded regions distinguish the range over which viability was at or above control (green) from the range in which cytotoxicity occurred (red). Viability was unchanged at 50 μM (96.7%), modestly but not significantly elevated at 75, 100 and 200 μM (115.4%, 116.7% and 112.0%), significantly elevated at 300 μM (160.3%), and reduced only at 400 and 500 μM (68.1% and 21.9%). Comparisons against the vehicle control were made by one-way ANOVA with Dunnett’s post hoc test: ns, not significant; * *p* < 0.05; **** *p* < 0.0001. Because the response is not monotonic, and because PFDA produced no measurable effect at the two concentrations used in combination (50 and 75 μM, effect fraction ≤ 0), the median-effect formalism underlying the Chou–Talalay method could not be applied to this compound pair.

PFDA produced no significant change in viability at 50 and 200 μM, a significant increase in absorbance at 75, 100, and 300 μM, and significant cytotoxicity at 400 and 500 μM (*p* < 0.0001), with a nominal cytotoxicity value of ~418 μM. Because PFDA produced a non-monotonic, hormetic dose–response rather than a conventional sigmoidal decline, this value—estimated from the 400–500 μM cytotoxic range alone—does not represent a classical IC_50_ and is interpreted with caution. Pilot experiments conducted in 6-well plate format—the format used for all subsequent adipogenesis, Oil Red O, and gene expression experiments—revealed progressive cell rounding and detachment within 48 h of exposure to PFDA concentrations ≥200 μM, a threshold substantially below that indicated by the 96-well WST-1 data. At working concentrations of 50 and 75 μM, PFDA did not alter cell morphology or adherence compared with the vehicle control (Figure 3). PFDA was therefore selected on the basis of morphological integrity in the 6-well format at these concentrations. Raw PFDA viability dose–response results are given in Figure 4.

#### 3.1.2. Effect of Curcumin + PFDA Combination on Cell Viability

All curcumin–PFDA combinations remained essentially non-cytotoxic, with viabilities of 100.7% (20 μM cu. + 50 μM PFDA), 95.6% (20 μM cu. + 75 μM PFDA), 92.6% (30 μM cu. + 50 μM PFDA), and 88.2% (30 μM cu. + 75 μM PFDA). In each case, viability was higher than for the corresponding curcumin monotherapy (90.9% and 85.6% at 20 and 30 μM), indicating that co-exposure to PFDA partially offset the mild cytotoxicity of curcumin. This was quantified using two independent reference models. Taking the single-agent effects measured on the same plate (curcumin: −9.05 and −14.39 percentage points at 20 and 30 μM; PFDA: +4.41 and 0.00 percentage points at 50 and 75 μM) each combination was slightly more viable than either reference model predicted, with mean interaction terms of +6.4, +5.4, +4.1 and +4.2 percentage points under Bliss independence for the 20 + 50, 20 + 75, 30 + 50 and 30 + 75 combinations. These terms were positive in direction across all four combinations, but none differed significantly from zero across the three biological replicates (*p* = 0.16 to 0.64), and a two-way ANOVA on the full curcumin by PFDA design confirmed significant main effects of curcumin (*p* = 0.0005) and PFDA (*p* = 0.019) with no significant interaction between them (*p* = 0.85). The higher viability of the combinations relative to curcumin alone is therefore attributable to the independent proliferative effect of PFDA rather than to a demonstrable interaction between the two compounds. Because PFDA alone was not cytotoxic over the tested range and the combined effects were small (≤12% loss of viability), these interaction terms are interpreted as indicators of the direction of the viability-level interaction rather than as precise quantitative estimates.

### 3.2. Oil Red O Staining—Intracellular Lipid Accumulation

#### 3.2.1. Effect of Curcumin and PFDA on Lipid Accumulation

Oil Red O staining on Day 8 of differentiation showed that curcumin-treated cells exhibited a dose-dependent decrease in intracellular lipid accumulation relative to the DMSO control. Spectrophotometric quantification at 510 nm revealed a significant reduction in lipid content relative to the vehicle control (*p* < 0.001), with 65.5% and 68.1% inhibition at 20 μM and 30 μM curcumin, respectively. PFDA at 50 μM increased lipid accumulation to 115.6% of the vehicle control (*p* < 0.01), confirming its pro-adipogenic activity under these conditions.

#### 3.2.2. Effect of Curcumin + PFDA Combination on Lipid Accumulation

**Figure 5:**
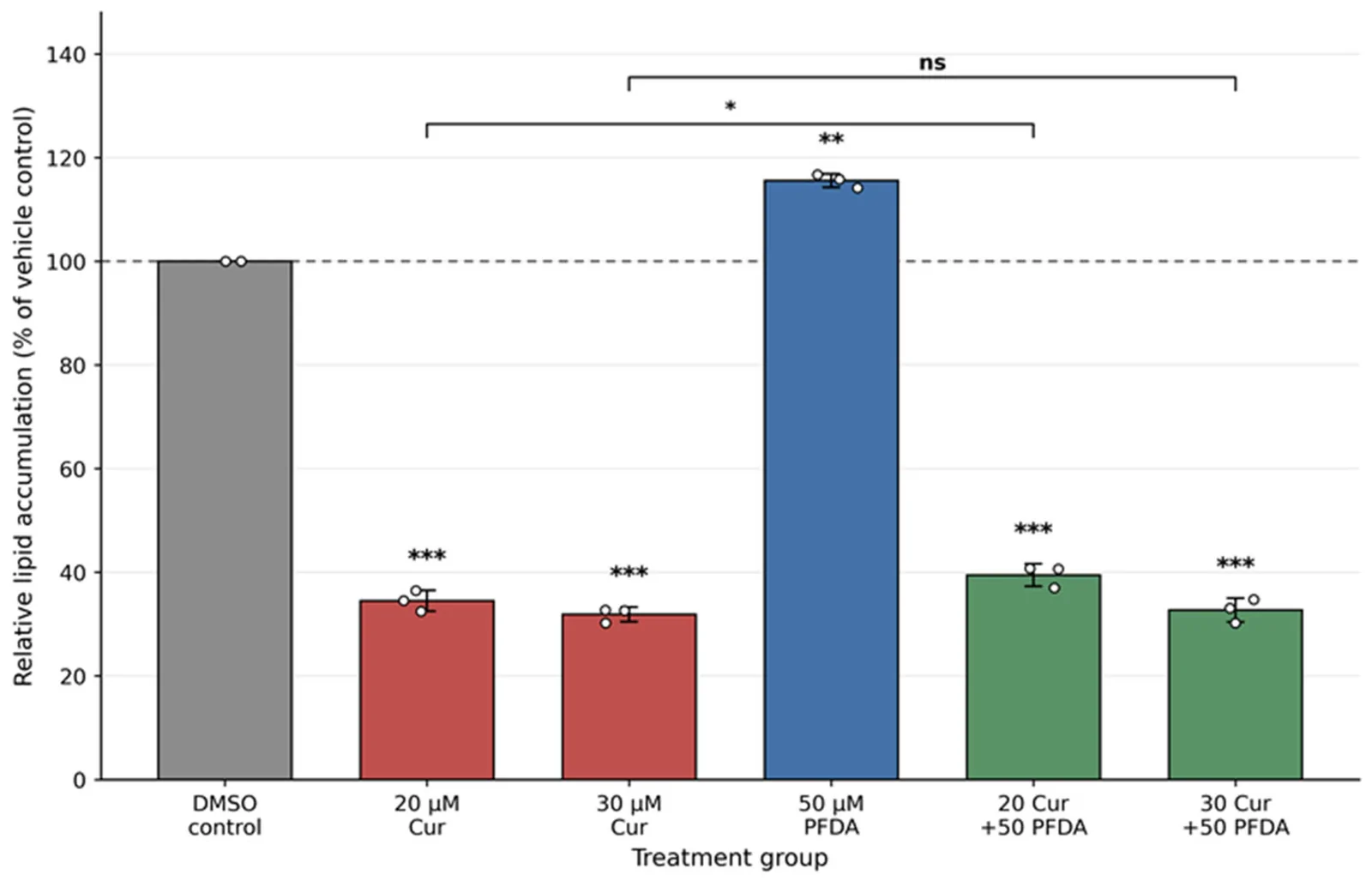
Effect of curcumin, PFDA and their combination on intracellular lipid accumulation in 3T3-L1 adipocytes. Lipid accumulation was quantified by Oil Red O staining on Day 8 of differentiation and normalized within each biological replicate to its own DMSO vehicle control (dashed line, 100%). Bars represent mean ± SD of three independent biological replicates (*n* = 3); open circles show the individual replicate values. Comparisons against the vehicle control were made by one-sample *t*-test: ** *p* < 0.01; *** *p* < 0.001. Brackets indicate paired comparisons between each curcumin monotherapy and its corresponding combination: ns, not significant; * *p* < 0.05.

**Figure 6:**
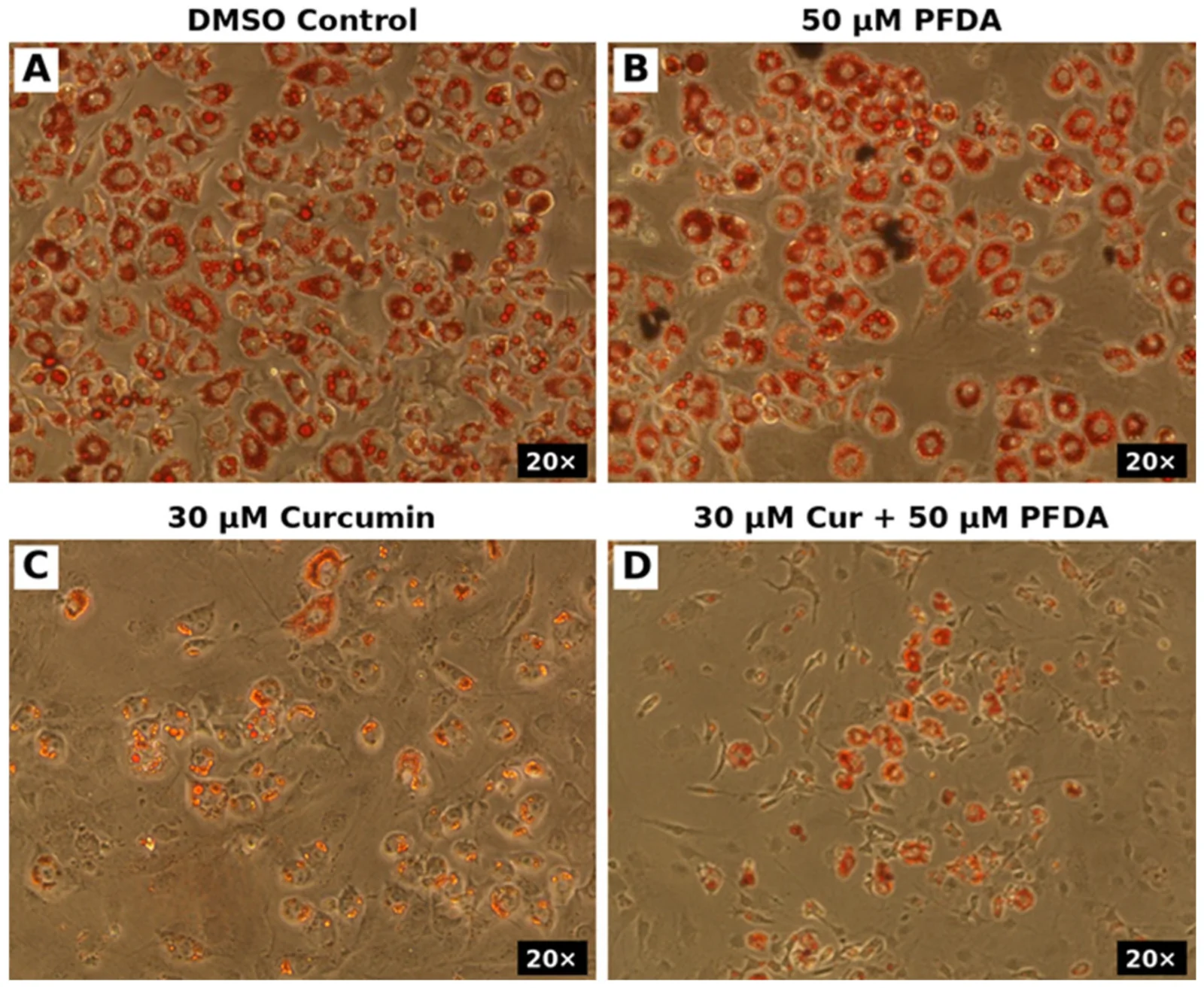
Oil Red O staining of 3T3-L1 adipocytes on Day 8 of differentiation (20× magnification) shows the following: (**A**) DMSO vehicle control; (**B**) 50 μM PFDA, increases lipid accumulation; (**C**) 30 μM curcumin, which markedly reduces lipid accumulation; and (**D**) a combination of 30 μM curcumin and 50 μM PFDA, where curcumin co-treatment suppresses lipid accumulation to levels similar to those observed with curcumin alone, despite the presence of PFDA. Red staining signifies the presence of intracellular neutral lipid droplets. The images shown are representative of the quantitative results presented in Figure 5.

Co-treatment with 20 μM curcumin + 50 μM PFDA and 30 μM curcumin + 50 μM PFDA reduced lipid accumulation to mean absorbance values of 0.108 and 0.089 at 510 nm, respectively, falling below the PFDA-alone group (0.317; control 0.274) and approaching the levels observed with curcumin alone (*p* < 0.001). Relative to the differentiation control, these combinations produced 60.5% and 67.3% inhibition for the 20 μM and 30 μM curcumin combinations, respectively (Figure 5). Functional lipid quantification by Oil Red O was performed for the 50 μM PFDA combination groups because 50 μM was the PFDA concentration confirmed to be sub-cytotoxic in the WST-1 assay and used throughout the combination and gene-expression experiments, so that the phenotypic and transcriptional endpoints refer to the same exposure; the 75 μM PFDA combinations were characterized at the transcriptional level only. Phenotypic conclusions regarding lipid accumulation in the combination groups are accordingly restricted to the 50 μM PFDA combinations (Figure 6).

### 3.3. RT-qPCR Gene Expression Analysis

Fold change was calculated relative to the DMSO control using the 2^−ΔΔCt^ method, with *Gapdh* as the reference gene.

#### 3.3.1. *Pparg* mRNA Expression

**Figure 7:**
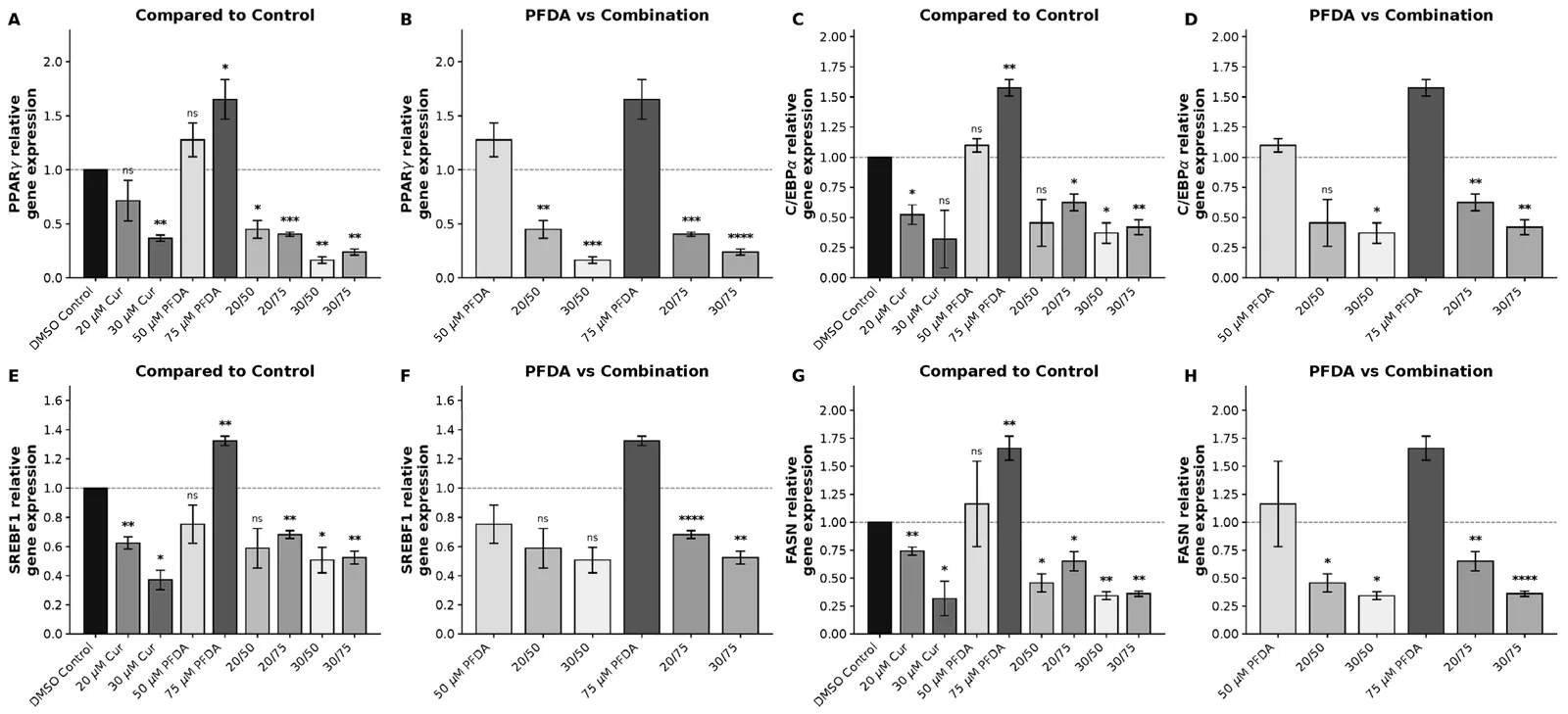
Effects of curcumin, PFDA, and their combinations on the mRNA expression of adipogenic and lipogenic genes in 3T3-L1 adipocytes. Relative gene expression was determined by RT-qPCR using the 2^−ΔΔCt^ method with *Gapdh* as the reference gene. On the concentration axis for combination groups, the first number corresponds to curcumin and the second to PFDA (i.e., 20/50). For each gene, the left panel shows expression relative to the DMSO control and the right panel the comparison between PFDA-only and curcumin + PFDA combination groups. Because 2^−ΔΔCt^ values are log-normally distributed, statistical testing was performed on log2-transformed fold changes: one-sample *t*-tests against a null value of 0 (fold change = 1) for comparisons with the DMSO control, and two-sample *t*-tests for the PFDA-versus-combination comparisons. (**A**,**B**) *Pparg*; (**C**,**D**) *Cebpa*; (**E**,**F**) *Srebf1*; (**G**,**H**) *Fasn*. Bars represent the mean ± SD of three independent biological replicates (*n* = 3), with technical replicates averaged within each biological replicate. The dashed line indicates control expression (fold change = 1). ns = not significant; * *p* < 0.05; ** *p* < 0.01; *** *p* < 0.001; **** *p* < 0.0001.

Curcumin at 30 μM significantly downregulated *Pparg* expression (0.365-fold; *p* = 0.002), whereas PFDA at 75 μM produced significant upregulation (1.652-fold; *p* = 0.016); neither 20 μM curcumin (0.714-fold) nor 50 μM PFDA (1.277-fold) alone produced a statistically significant change. All four combination groups significantly inhibited *Pparg* relative to the control: 20 μM cu. + 50 μM PFDA reduced expression to 0.448-fold (*p* = 0.019), 20 μM cu. + 75 μM PFDA to 0.404-fold (*p* < 0.001), 30 μM cu. + 50 μM PFDA to 0.164-fold (*p* = 0.004; the strongest *Pparg* suppression observed in this study), and 30 μM cu. + 75 μM PFDA to 0.237-fold (*p* = 0.002). Direct comparison of the PFDA-only and combination groups confirmed that curcumin significantly reversed PFDA-induced *Pparg* upregulation across all combinations (*p* = 0.003, *p* < 0.001, *p* < 0.001, and *p* < 0.0001, respectively) (Figure 7A,B).

#### 3.3.2. *Cebpa* mRNA Expression

Curcumin significantly reduced *Cebpa* expression at 20 μM (0.524-fold; *p* = 0.019), whereas the reduction at 30 μM (0.320-fold; *p* = 0.101) did not reach significance owing to inter-replicate variability. PFDA at 75 μM significantly increased *Cebpa* (1.575-fold; *p* = 0.003), while 50 μM PFDA (1.098-fold) had no significant effect. Compared with the control, significant inhibition was observed at 20 μM cu. + 75 μM PFDA (0.624-fold; *p* = 0.017), 30 μM cu. + 50 μM PFDA (0.371-fold; *p* = 0.018), and 30 μM cu. + 75 μM PFDA (0.419-fold; *p* = 0.009) groups, whereas the 20 μM cu. + 50 μM PFDA group (0.455-fold; *p* = 0.098) did not reach significance. In the PFDA-versus-combination comparison, curcumin significantly suppressed *Cebpa* for the 30 μM cu. + 50 μM PFDA, 20 μM cu. + 75 μM PFDA, and 30 μM cu. + 75 μM PFDA groups (*p* = 0.012, *p* = 0.002, and *p* = 0.002, respectively), but not for the 20 μM cu. + 50 μM PFDA group (*p* = 0.081) (Figure 7C,D).

#### 3.3.3. *Srebf1* mRNA Expression

*Srebf1* was significantly downregulated by curcumin at both 20 μM (0.624-fold; *p* = 0.006) and 30 μM (0.371-fold; *p* = 0.010). An amount of 50 μM PFDA did not significantly change *Srebf1* (0.752-fold), whereas 75 μM PFDA produced a modest but significant increase (1.324-fold; *p* = 0.003), the weakest induction among the four genes and absent at the 50 μM dose. Because *Srebf1* and *Pparg* are reciprocally regulated, this dose-restricted change is not by itself informative about which arm of the adipogenic program PFDA engages, and its interpretation is considered in the Discussion. Among the combination groups, significant inhibition relative to the control was observed for 20 μM cu. + 75 μM PFDA (0.681-fold; *p* = 0.004), 30 μM cu. + 50 μM PFDA (0.508-fold; *p* = 0.021), and 30 μM cu. + 75 μM PFDA (0.524-fold; *p* = 0.006), whereas 20 μM cu. + 50 μM PFDA (0.588-fold; *p* = 0.056) did not reach significance. In the PFDA-versus-combination comparison, curcumin significantly suppressed *Srebf1* at 75 μM PFDA (*p* < 0.001 and *p* = 0.002 for the 20 and 30 μM combinations) but not at 50 μM PFDA (*p* = 0.219 and *p* = 0.055) (Figure 7E,F).

#### 3.3.4. *Fasn* mRNA Expression

Curcumin significantly downregulated *Fasn* at both 20 μM (0.742-fold; *p* = 0.009) and 30 μM (0.317-fold; *p* = 0.049), whereas 75 μM PFDA significantly upregulated it (1.662-fold; *p* = 0.005); 50 μM PFDA produced no significant change (1.164-fold; *p* = 0.646). All four combination groups significantly suppressed *Fasn* relative to the control (20 μM cu. + 50 μM PFDA: 0.458-fold, *p* = 0.017; 30 μM cu. + 50 μM PFDA: 0.343-fold, *p* = 0.003; 20 μM cu. + 75 μM PFDA: 0.651-fold, *p* = 0.030; 30 μM cu. + 75 μM PFDA: 0.360-fold, *p* = 0.001). PFDA-only versus combination comparisons confirmed significant reversal of PFDA-induced *Fasn* upregulation in all four groups (*p* = 0.031, *p* = 0.022, *p* = 0.002, and *p* < 0.0001, respectively) (Figure 7G,H).

## 4. Discussion

In this study, curcumin opposed PFDA-induced adipogenesis at multiple levels of the transcriptional program that governs 3T3-L1 differentiation, assessed here as the mRNA expression of *Pparg*, *Cebpa*, *Srebf1* and *Fasn*, which encode PPARγ, C/EBPα, SREBP-1 and FASN, respectively [5]. The following sections interpret these findings in the order of the experimental workflow, beginning with cytotoxicity and dose selection.

### 4.1. Interpreting the Viability-Level Interaction

The 3T3-L1 preadipocyte cell line was chosen as the experimental model because of its well-established utility in adipogenesis research and its growing use as a screening platform for environmental obesogens [6]. The curcumin IC_50_ of 56.15 μM aligns with Zhao et al. [26], who reported cytotoxic effects at concentrations ≥50 μM in murine cell lines. Both 20 and 30 μM fall below the cytotoxic threshold while retaining pharmacological activity relevant to adipogenic endpoints. For PFDA, the morphological cytotoxicity observed at ≥200 μM in the 6-well format indicates that the nominal 96-well cytotoxicity estimate (~418 μM) overestimates PFDA tolerance under the conditions used for the differentiation experiments; working concentrations were therefore selected conservatively. Because the in vitro concentrations used here (50–500 μM) exceed typical human serum levels (0.2 ng/mL, ~0.39 nM) by approximately 100,000- to 1,000,000-fold, they should be regarded as mechanistic probes that compensate for short exposure durations (24 to 72 h) rather than physiologically realistic exposures [15,16].

Several considerations place these concentrations in context. First, acute in vitro exposures (24 to 72 h during differentiation) cannot reproduce the decades-long chronic accumulation that occurs in vivo; elevated concentrations are therefore routinely employed as a mechanistic probe to elicit measurable transcriptional and phenotypic responses within a short window, rather than to model systemic exposure. Second, and importantly, tissue distribution does not close this gap. Perfluoroalkyl acids are not lipophilic in the conventional sense: they bind serum albumin and fatty-acid-binding proteins rather than partitioning into neutral lipid, and therefore accumulate in protein-rich compartments such as liver, kidney and lung, with reported human tissue-to-serum ratios on the order of 1.3:1 and no preferential enrichment in adipose tissue. We therefore do not invoke tissue partitioning to justify the concentrations used; the difference between them and population serum levels remains a genuine limitation of the present design [17,18]. Third, the concentrations used here are consistent with the established in vitro PFAS-adipogenesis literature: Watkins et al. [27] examined PFOA and PFNA at 5–100 μM, PFOS at 50–300 μM, and PFHxS at 40–250 μM in 3T3-L1 cells, and a proteomic screen of ten PFAS, including PFDA, in the same model employed comparable micromolar exposures (0.25–25 μM) [28]; comparable ranges have been applied to characterize PFAS effects on hepatic lipid metabolism [29]. Importantly, the combination concentrations that produced the functional adipogenic and transcriptional findings (20, 30 μM curcumin with 50, 75 μM PFDA) were sub-cytotoxic and preserved cell morphology, indicating that the reported effects reflect genuine responses in viable adipocytes rather than artifacts of overt toxicity. These concentrations should therefore be interpreted as a mechanistic probe of the adipogenic mode of action of PFDA, not as a surrogate for human exposure levels. Accordingly, the present findings do not support quantitative inference about human risk at environmentally relevant exposures. Establishing whether the transcriptional program described here operates at chronically relevant concentrations will require longer, lower-dose exposures, ideally in human primary or stem-cell-derived adipocytes.

The format-dependent discrepancy arises mechanistically from the physicochemical properties of PFDA, a long-chain perfluorocarbon surfactant that strongly adsorbs to hydrophobic surfaces such as tissue culture-grade polystyrene [30]. Because 96-well plates have a higher surface-area-to-volume ratio, a proportionally greater amount of PFDA adsorbs to the plastic, reducing bioavailable solution-phase concentrations and attenuating apparent cytotoxicity in the WST-1 readout compared to the 6-well format, where a greater fraction of PFDA remains in solution and exerts membrane-level effects at equivalent nominal concentrations. Bjork and Wallace [31] used 100 μM PFDA in HepG2/C3A cells and reported similar cytotoxicity profiles, so the selected working concentrations of 50 and 75 μM PFDA fall within the same range. Inter-laboratory differences in cell passage number, media composition, and compound source—a known source of variation in perfluorinated compound bioassays [31]—likely explain the dose discrepancy compared to Wang et al. [9], who applied 1–10 μM concentrations. This format dependence reconciles the apparently divergent readouts at 75–100 μM PFDA: the elevated WST-1 absorbance in 96-well plates reflects reduced bioavailable compound and a mild metabolic/proliferative response, whereas in the 6-well format the same nominal doses produced morphological stress and the decline in lipid accumulation observed by Oil Red O.

At the viability level, PFDA and curcumin acted independently, with PFDA partially offsetting the cytotoxicity of curcumin, with positive interaction terms for all four combinations; every combination was in fact less cytotoxic than curcumin alone. This follows directly from the behaviour of PFDA as a single agent, which was not cytotoxic over the tested range and instead produced a mild hormetic response (viability ≥96% up to 300 μM), so that a sub-cytotoxic, proliferative concentration of PFDA partially offsets curcumin’s modest cytotoxicity. Because PFDA lacked a monotonic cytotoxic dose–response, these interaction terms are interpreted as evidence for the direction of the interaction rather than its magnitude. This attenuation of curcumin-mediated cytotoxicity at sub-IC_50_ PFDA concentrations likely arises from adaptive cellular responses to sub-lethal PFAS exposure, such as modulation of membrane dynamics, activation of antioxidant defense pathways, or *Pparg*-mediated pro-survival signaling, that partially counteract curcumin’s proliferative inhibition without engaging the adipogenic regulatory circuitry central to its antiadipogenic activity [32]. Critically, interaction estimates derived from cytotoxicity data are inherently endpoint-specific: the behaviour of the two compounds at the level of proliferative inhibition carries no predictive value for their combined effect on adipogenic differentiation, lipid accumulation, or gene expression [22]. The central interpretive finding of this study is the dissociation between these viability-level effects and the antiadipogenic efficacy demonstrated in the functional and transcriptional analyses that follow.

### 4.2. Quantifying the Antiadipogenic Offset

Our Oil Red O data replicate the finding of Ejaz et al. that adipogenesis in 3T3-L1 cells is inhibited in a concentration-dependent manner, and extend these observations to in vivo settings, demonstrating reduced adipose tissue formation in curcumin-supplemented mice. Although the difference in lipid inhibition between the two curcumin concentrations was modest at 2.2 percentage points, the gene expression data support dose-dependent antiadipogenic activity; 30 μM curcumin consistently produced stronger transcriptional suppression of *Pparg*, *Cebpa*, and *Fasn* than 20 μM curcumin, providing mechanistic corroboration of concentration-dependent activity across the working dose range [25,33].

Whereas curcumin suppressed lipid accumulation, PFDA exerted the opposite effect, consistent with its classification as a peroxisome proliferator and its established capacity to activate PPARγ signaling, thereby promoting adipocyte differentiation and lipid deposition. In the present experiments PFDA increased lipid accumulation to 115.6% of the vehicle control at 50 μM (*p* < 0.01). This non-monotonic pattern aligns with two non-exclusive mechanisms: a lipogenic ceiling, in which PPARγ-mediated adipogenic activation nears saturation at lower concentrations, and incipient cytotoxicity at higher doses, which diminishes the pool of metabolically intact adipocytes capable of lipid accumulation, consistent with the morphological cytotoxicity observed at elevated PFDA concentrations (Figure 3). These observations align with Yamamoto et al. [34], who reported that the structurally related compound PFOA increases triglyceride levels and adipogenic gene expression in 3T3-L1 cells, and with Wang et al. [9], who confirmed that PFDA promotes triglyceride accumulation in the same cell line; recent epidemiological and experimental evidence linking perfluoroalkyl substance exposure to adipogenic dysregulation and obesity-related metabolic outcomes supports these findings [35].

Curcumin retained substantial antiadipogenic efficacy even under conditions of PFDA co-exposure. Both 50 μM PFDA combination groups reduced lipid accumulation to near-monotherapy levels (60.5% and 67.3% inhibition for the 20 μM and 30 μM curcumin combinations, respectively), markedly below PFDA alone. The slight reduction in curcumin’s antiadipogenic efficacy within combination groups compared to monotherapy, roughly 5 percentage points at 20 μM and under 1 percentage point at 30 μM, results from the competing pro-adipogenic stimulus of PFDA rather than from the viability-level behavior described above. To quantify this offset explicitly, we distinguish the inherent antiadipogenic capacity of curcumin from its residual capacity once PFDA-driven lipogenesis has been counteracted. Referenced to the differentiation control, curcumin alone inhibited lipid accumulation by 65.5% (20 μM) and 68.1% (30 μM), whereas the corresponding combinations achieved 60.5% and 67.3%; the cost imposed by PFDA co-exposure is therefore 5.0 and 0.8 percentage points, respectively. This difference was statistically significant for the 20 μM pairing (*p* = 0.013) but not for the 30 μM pairing (*p* = 0.34). Referenced instead to the PFDA-stimulated baseline, which is the lipid load curcumin must actually overcome (115.6% of control), the net inhibition attributable to curcumin is 65.9% and 71.7% for the 20 and 30 μM combinations, so that net efficiency against the elevated baseline equals or exceeds the inherent inhibition measured in the absence of the obesogen. Expressed a third way, residual lipid in the combination groups exceeded that in the corresponding curcumin-only groups by only 14.4% (20 μM) and 2.6% (30 μM). Although PFDA raised lipid content to 115.6% of control, both combinations finished 60.5% and 67.3% below control, indicating that curcumin not only neutralized the PFDA-induced increment but suppressed lipid accumulation well beyond the untreated differentiation baseline. At 30 μM curcumin the obesogenic stimulus was thus entirely offset, whereas at 20 μM a small but measurable erosion of efficacy remained, defining a concentration-dependent limit to the protective effect. The near-complete preservation of curcumin’s lipid-accumulation inhibition in both 50 μM PFDA combination groups demonstrates a functionally resilient antiadipogenic effect, as shown in Figure 6, providing the basis for the transcriptional analyses that follow.

### 4.3. RT-qPCR mRNA Expression

#### 4.3.1. *Pparg* as the Dominant Regulatory Node

The *Pparg* expression data provide a transcriptional basis for the functional outcomes observed in Oil Red O quantification while resolving the apparent contradiction between the viability-level effects and preserved antiadipogenic efficacy. The 75 μM PFDA upregulation (1.652-fold) is consistent with PFDA’s established role as a PPARγ ligand and peroxisome proliferator that activates PPARγ-dependent transcriptional programs in adipocytes. The absence of significant *Pparg* regulation by 50 µM PFDA, despite its pro-adipogenic effect, suggests that PFDA’s lipid-accumulation-promoting activity at this concentration may reflect a sub-threshold level of *Pparg* activation insufficient to alter steady-state mRNA abundance statistically, while still exerting measurable downstream effects on lipid metabolism. The combination of 30 μM curcumin and 50 μM PFDA produced the most potent *Pparg* suppression in the study (0.164-fold), substantially exceeding the suppression from 30 μM curcumin alone (0.365-fold), likely reflecting curcumin’s direct inhibition of PPARγ transcription together with PFDA’s sub-threshold modulation of the transcriptional microenvironment [3]. Although 20 μM curcumin alone produced no significant change, the same dose significantly suppressed *Pparg* below control within the combination; this reflects a transcriptional threshold shift in which the higher baseline set by PFDA-driven PPARγ upregulation allows even modest curcumin input to yield a detectable net suppression, rather than any change in curcumin’s intrinsic mechanism. Mechanistically, curcumin suppresses PPARγ by directly binding its ligand-binding domain, by modulating upstream regulators including Wnt/β-catenin, by activating AMPK signaling, and by blocking C/EBPβ-mediated PPARγ-induction pathways, which remain sensitive to curcumin despite concurrent PPARγ activation by PFDA [36,37]. The consistent *Pparg* suppression across both combinations identifies PPARγ inhibition as the primary mechanism of curcumin’s preserved antiadipogenic efficacy under PFDA co-exposure.

#### 4.3.2. *Cebpa* and the Reinforcement of the Adipogenic Program

The *Cebpa* response paralleled that of *Pparg*, reflecting the well-established positive feedback loop between these two master adipogenic transcription factors, in which each reinforces the other’s expression during terminal adipocyte differentiation [38]. The parallel dose thresholds (significant effects only at 30 μM curcumin and 75 μM PFDA) indicate coordinate regulation of these two transcription factors. Kim et al. reported that 10 and 30 μM curcumin significantly reduced *Pparg* and *Cebpa* mRNA levels in 3T3-L1 cells, attributing this effect to modulation of mitotic clonal expansion during early differentiation; the concentration–response profile observed here aligns with these findings [11]. The dose-dependent pattern—complete suppression below control at 50 μM PFDA but only partial attenuation at 75 μM PFDA—indicates that curcumin’s inhibitory effect on *Cebpa* is sufficient to counteract PFDA’s pro-adipogenic influence at the lower concentration, whereas high-dose PFDA maintains *Cebpa* expression via pathways that curcumin, at the concentrations tested, cannot fully disrupt. Consistent with this, curcumin significantly reduced *Cebpa* in both the 30 μM cu. + 50 μM PFDA (0.371-fold, *p* = 0.018) and the 30 μM cu. + 75 μM PFDA (0.419-fold, *p* = 0.009) groups, indicating a robust inhibitory effect across both PFDA doses. A potential, though untested, mechanism involves the upstream regulators C/EBPβ and C/EBPδ, which can induce C/EBPα independently of PPARγ; since these factors were not assessed in this study, this interpretation remains hypothetical and requires direct verification. Considered alongside the *Pparg* data, these findings indicate that curcumin’s antiadipogenic transcriptional effect extends across the PPARγ/C/EBPα positive feedback axis, and the completeness of *Cebpa* suppression depends on the prevailing PFDA concentration.

#### 4.3.3. Limited Engagement of the SREBP-1c Arm

Unlike the patterns observed for *Pparg* and *Cebpa*, PFDA produced only a weak, dose-restricted change in *Srebf1* expression, reaching significance only at the 75 μM dose. We interpret this divergence cautiously: the induction of *Srebf1* at 75 μM (1.324-fold) was smaller than that of *Pparg* (1.652-fold), *Cebpa* (1.575-fold) and *Fasn* (1.662-fold) and was absent at 50 μM, but the four transcripts were not formally compared with one another, no additional markers of the SREBP-1c arm were measured, and no protein-level data are available. Because *Srebf1* and *Pparg* are reciprocally regulated, a modest *Srebf1* change may itself be secondary to PPARγ activation. The pattern is therefore compatible with, but does not establish, a pro-adipogenic transcriptional drive in the 3T3-L1 model that primarily operates through the classical PPARγ/C/EBPα axis, with only limited engagement of the SREBP-1c-mediated lipogenic arm. This pathway specificity aligns with the established regulatory architecture of *Srebf1*, since insulin/PI3K/Akt signaling and liver X receptor (LXR) activation—rather than direct PPARγ ligand-mediated transactivation—primarily govern its transcription [39]. Given that PFDA is a PPARγ ligand and peroxisome proliferator, it would not be expected to activate the LXR or insulin-responsive elements that regulate *Srebf1* transcription under basal differentiation conditions, a prediction corroborated by the present data. The broader dose–response at *Srebf1* (significant suppression by curcumin at both 20 and 30 μM) indicates that the lipogenic regulatory axis is more sensitive to curcumin’s inhibitory activity than the classical PPARγ/C/EBPα program [40], suggesting that curcumin engages at least two independent antiadipogenic axes: a PPARγ/C/EBPα transcriptional axis requiring ≥30 μM curcumin for significant suppression, and an *Srebf1*-mediated lipogenic axis accessible at 20 μM curcumin through AMPK/PI3K-dependent mechanisms. The absence of a significant combination effect at 50 µM PFDA is not evidence of pathway convergence; it may equally reflect limited statistical power, a floor effect, or true non-additivity. Although PFDA and curcumin might target shared regulatory nodes within the AMPK/PI3K axis at this concentration, the present data cannot confirm this possibility; we therefore advance it as a hypothesis requiring direct testing [41]. Nonetheless, the finding that both combination groups suppress *Srebf1* to a level significantly below that of the differentiation control confirms that curcumin retains its inhibitory activity on the lipogenic axis under PFDA co-exposure, though the precise mechanistic basis remains to be established.

#### 4.3.4. *Fasn* as the Downstream Lipogenic Effector

Fatty acid synthase (FASN), the rate-limiting enzyme in de novo lipogenesis, exhibited a monotherapy response pattern consistent with that observed for *Pparg* and *Cebpa*. This convergent monotherapy behavior, however, masks a key difference in lipogenesis. Although FASN is canonically regulated by SREBP-1c as its primary lipogenic transcriptional driver [39], PFDA significantly induced *Fasn* expression without producing a statistically significant change in *Srebf1*. The induction of *Fasn* aligns with the lipogenic upregulation reported by Wang et al. [9]; however, *Srebf1* was not similarly upregulated in our dataset, contrary to what Wang et al. [9] observed, a discrepancy that may reflect differences in exposure concentration, treatment duration, or differentiation stage. The observed dissociation—*Fasn* induction with only a weak *Srebf1* change—indicates that PFDA induces lipogenic enzyme expression through an *Srebf1*-independent mechanism. FASN contains peroxisome proliferator-activated receptor response elements (PPREs) within its promoter, which has also been reported to be regulated by PPARγ. C/EBPα has similarly been shown to contribute to FASN regulation. The data therefore support the interpretation that PFDA drives *Fasn* expression primarily through its *Pparg*/*Cebpa* agonism rather than the SREBP-1c pathway, providing retrospective mechanistic coherence to the *Srebf1* null result and identifying the *Pparg*/*Cebpa* axis as the dominant transcriptional conduit through which PFDA exerts its pro-lipogenic effect at the enzymatic level. Curcumin’s reversal of PFDA-induced *Fasn* upregulation was significant across all four combinations (*p* ≤ 0.031), confirming that the transcriptional changes in *Pparg* and *Cebpa* translate into reduced lipogenic enzyme capacity. Our downregulation data of *Pparg* and *Cebpa* is consistent with Ahn et al. [36], who showed that curcumin suppresses adipogenic differentiation in 3T3-L1 cells by restoring Wnt/β-catenin signaling and inhibiting MAPK activation—upstream events that limit induction of the PPARγ/C/EBPα program. The accompanying reduction in *Srebf1* and *Fasn* in our data is in line with Zhao et al. [42], who characterized curcumin as a functional FASN inhibitor that blocks differentiation and lipid accumulation. *Fasn* mRNA suppression in curcumin-treated and co-treated groups aligns directionally with the reduction in intracellular lipid accumulation quantified by Oil Red O, providing an integrated mechanistic account that connects transcriptional suppression of the master adipogenic program, through enzymatic inhibition of lipogenic capacity, to the observed phenotype of reduced lipid accumulation [43].

#### 4.3.5. Four-Gene Mechanistic Synthesis

Transcriptional and enzymatic data on *Pparg*, *Cebpa*, *Srebf1*, and *Fasn* demonstrate that the curcumin and PFDA interaction disrupts 3T3-L1 adipocyte differentiation through a multi-level mechanism. At 75 μM, PFDA significantly upregulates *Pparg* transcription, whereas curcumin consistently suppresses it across all four combination groups, indicating it as the dominant regulatory node. At the secondary level, *Cebpa* is a coupled target that is suppressed in parallel with *Pparg*; its expression fails to drop below control levels at 75 μM PFDA, suggesting that high-dose PFDA sustains *Cebpa* expression via mechanisms, potentially involving PPARγ-independent regulators such as C/EBPβ and C/EBPδ, that curcumin, at the concentrations tested, cannot fully antagonize, as previously discussed. PFDA’s pro-adipogenic program level is dominated, at the transcriptional level, by upregulation of the PPARγ/C/EBPα program rather than the SREBP-1c lipogenic pathway—a signature consistent with, though not by itself proving, PPARγ/C/EBPα-driven adipogenesis, as evidenced by markedly weaker *Srebf1* induction despite significant induction of *Cebpa* and *Fasn*. At lower dose thresholds, curcumin independently suppresses *Srebf1*, likely through AMPK/PI3K-mediated mechanisms, which extends its inhibitory effects beyond the PPARγ/C/EBPα program. Suppression of *Fasn* at the effector level confirms that upstream transcriptional changes translate into reduced lipogenic enzyme capacity. Curcumin’s capacity to inhibit multiple adipogenic targets makes it a candidate antiadipogenic agent for further investigation in PFAS-associated metabolic disruption. Stratifying this activity by obesogen concentration shows that the transcriptional efficiency of curcumin was maintained, and for most targets increased, at the higher PFDA concentration. Expressed as the percentage suppression of each transcript relative to its corresponding PFDA-alone group, 30 μM curcumin suppressed *Pparg* by 87.2% at 50 μM PFDA and 85.7% at 75 μM PFDA, *Cebpa* by 66.2% and 73.4%, *Fasn* by 70.5% and 78.3%, and *Srebf1* by 32.4% and 60.4%, respectively; the corresponding values for 20 μM curcumin were 64.9% and 75.5% for *Pparg*, 58.6% and 60.4% for *Cebpa*, 60.7% and 60.8% for *Fasn*, and 21.8% and 48.6% for *Srebf1*. Two patterns emerge. First, curcumin does not lose transcriptional potency as the obesogenic challenge increases: for *Pparg* its suppression is near-maximal at both PFDA concentrations, indicating that this node is efficiently targeted across the tested range. Second, the gain in efficiency at 75 μM PFDA is most pronounced for *Srebf1*, which is also the only transcript significantly induced by PFDA at the higher concentration alone; curcumin therefore exerts its clearest incremental effect precisely where PFDA engages the lipogenic arm of the program. Taken together, these stratified data indicate that the antiadipogenic transcriptional response to curcumin is not attenuated by a higher obesogen burden within the concentration range examined, although this conclusion rests on transcriptional endpoints alone at 75 μM PFDA.

## 5. Conclusions

This study demonstrates that curcumin retains potent, multi-target antiadipogenic activity in 3T3-L1 cells co-exposed to the environmental obesogen PFDA, even though PFDA partially offsets its effect on cell viability. Functional confirmation at the level of lipid accumulation was obtained for the 50 μM PFDA combinations, which represent the maximal adipogenic challenge; efficacy at 75 μM PFDA is supported by transcriptional data alone. This applies in cytotoxicity. The dissociation between endpoints represents the key conceptual advance: combination effects on cell viability cannot predict interactions in adipogenesis, demonstrating that interactions between obesogens and protective phytochemicals must be characterized at the relevant biological endpoint rather than inferred solely from cytotoxicity-based indices. Curcumin operates through a tiered transcriptional program in which *Pparg* suppression is the dominant node, and this activity is concentration-resilient: relative to the corresponding PFDA-alone groups, transcriptional suppression by curcumin was maintained or enhanced at 75 μM PFDA compared with 50 μM PFDA across all four genes. This is reinforced by *Cebpa* co-suppression and complemented by *Srebf1* inhibition via a putative AMPK/PI3K-mediated axis, with confirmation at the lipogenic effector *Fasn*. Because PFDA induced *Srebf1* only weakly and only at the higher concentration, and because the transcripts were not formally compared, no additional markers of the SREBP-1c arm were assessed and no protein-level data were obtained; the following remains a possible interpretation of the observed changes rather than an established mechanism. Because PFDA only weakly induces *Srebf1*, the obesogen’s pro-adipogenic drive appears to originate predominantly, though not exclusively, from the PPARγ/C/EBPα axis rather than primarily from the SREBP-1c cascade. Chronic PFAS exposure is now consistently associated with metabolic dysfunction, and the present findings support curcumin as a biologically plausible dietary countermeasure against environmentally driven metabolic disruption. We outline four specific steps for this translational path. First, the transcriptional signature described here should be tested in human adipocyte systems, using primary subcutaneous and visceral preadipocytes, the Simpson-Golabi-Behmel Syndrome (SGBS) cell strain, or mesenchymal stem cell-derived adipocytes, to establish whether PFDA engages the same program and whether curcumin opposes it in human cells; parallel testing of subcutaneous and visceral donors would also indicate whether the response is depot-specific. Second, the mechanism requires protein-level confirmation, minimally by immunoblotting for PPAR-γ, C/EBP-α and FASN, together with PPAR-γ reporter or competitive-binding assays to establish whether PFDA acts as a direct receptor agonist rather than through upstream signaling. Third, chronic in vivo studies are needed in which rodents receive PFDA at environmentally relevant, low doses over an extended period, with and without dietary curcumin supplementation, assessing adipose depot mass, adipocyte size distribution, adipogenic gene and protein expression, and systemic metabolic parameters; developmental exposure windows deserve particular attention given the detection of PFDA in cord blood and breast milk. Fourth, translation to a dietary intervention will require attention to the low oral bioavailability of curcumin, so that formulation strategies and achievable adipose-tissue concentrations are defined before any inference is drawn about dietary intake in humans. Taken together, these steps would establish whether the interaction described here has physiological relevance, warranting extension of this work to chronic exposures alongside human adipocyte and in vivo models.

## 6. Limitations

This study has several limitations. The findings are restricted to the murine 3T3-L1 cell line, and direct extrapolation to human adipogenesis is constrained by interspecies differences and the absence of systemic hormonal and metabolic inputs. Curcumin’s preserved antiadipogenic activity was functionally confirmed by Oil Red O only for the 50 μM PFDA combinations; the 75 μM combinations were assessed transcriptionally alone. Lipid accumulation was quantified spectrophotometrically as extracted Oil Red O absorbance, which reports total neutral lipid per well but not its distribution between cells. Morphometric measures such as lipid droplet area, droplet diameter distribution and the proportion of differentiated cells were not quantified, and the micrographs presented are illustrative rather than a source of quantitative data. Such measures would indicate whether the reduction in total lipid reflects fewer differentiated adipocytes, smaller droplets within a comparable number of cells, or both, and would strengthen the phenotypic characterization; image-based morphometry is planned for subsequent work. The PFDA concentrations exceed typical human serum levels and should be regarded as mechanistic probes rather than physiologically realistic exposures. Expression was normalized to *Gapdh*; although raw *Gapdh* Ct values showed low within-run variability (mean CV = 2.6%) with all groups processed in parallel, *Gapdh* expression can vary during adipocyte differentiation, and normalization to the geometric mean of multiple validated reference genes is preferable. Two features of the present design constrain the impact of this limitation. First, all groups, including the vehicle control, were harvested at a single fixed differentiation endpoint (Day 8) and processed in parallel, so stage-dependent variation in *Gapdh* expression, which arises principally when different differentiation timepoints are compared, does not act as a between-group confounder here. Second, any residual bias would be conservative with respect to the findings reported: if *Gapdh* were induced in parallel with adipogenesis, normalization would underestimate PFDA-driven upregulation of the target transcripts in the more adipogenic PFDA-alone groups, and would likewise underestimate the magnitude of curcumin-mediated suppression in the less adipogenic curcumin and combination groups. Reference-gene drift would therefore attenuate rather than exaggerate the reported effects. Multi-gene normalization is being incorporated into ongoing work [44]. Finally, the absence of protein-level validation, particularly for *Fasn*, limits the mechanistic conclusions drawn from mRNA data, and the hypothesized convergence of the *Srebf1* pathway at 50 μM PFDA warrants targeted investigation of AMPK/mTOR signaling.

## Data Availability

The raw data supporting the conclusions of this article will be made available by the authors on request.

## Abbreviations

The following abbreviations are used in this manuscript:

PFDA	Perfluorodecanoic acid
PPARγ	Peroxisome proliferator-activated receptor γ
C/EBPα	CCAAT/enhancer-binding protein α
SREBP-1	Sterol regulatory element-binding protein 1
FASN	Fatty acid synthase
PFAS	Perfluoroalkyl and polyfluoroalkyl substances
PFC	Perfluorinated compounds
DMSO	Dimethyl sulfoxide
IBMX	3-isobutyl-1-methylxanthine
INS	Insulin
DEX	Dexamethasone
FBS	Fetal bovine serum
DF	Differentiation medium
ORO	Oil red O
RT-qPCR	Real time quantitative polymerase chain reaction
cu.	Curcumin
PPREs	Peroxisome proliferator response elements
PBS	Phosphate buffer saline
